# Magnetic resonance imaging with pathological correlation in a case of mantle cell lymphoma of the parotid gland: a case report

**DOI:** 10.1186/1752-1947-4-99

**Published:** 2010-03-30

**Authors:** Mayia Pilavaki, Anastasia Athanasiadou, Fotis Iordanidis, Thrasivulos Karakozoglou, Panagiotis Palladas

**Affiliations:** 1 Department of Radiology, General Hospital G Papanikolaou, Thessaloniki, Greece; 2 Department of Haematology, General Hospital G Papanikolaou, Thessaloniki, Greece; 3 Laboratory of Pathology, General Hospital G Papanikolaou, Thessaloniki, Greece

## Abstract

**Introduction:**

Mantle cell lymphoma is a rare non-Hodgkin's lymphoma. It is a subtype of B-cell lymphoma with frequent involvement of the bone marrow and the gastrointestinal tract. Isolated parotid gland involvement seldom occurs. Here we report an unusual case of isolated infiltration of the parotid gland by mantle cell lymphoma. The aim of our study is to correlate magnetic resonance imaging findings with the histological features of the disease. To the best of our knowledge, no similar radiological findings of mantle cell lymphoma have been published before.

**Case presentation:**

A 72-year-old Caucasian woman presented with a painful left parotid enlargement. She was diagnosed with mantle cell lymphoma involving the left submandibular gland seven years prior to presentation. Her whole body CT scan showed the absence of pathologically enlarged lymph nodes. However, a magnetic resonance imaging showed enlargement of her left parotid gland and an abnormal parenchyma with mixed-type solid and cystic lesions. A biopsy of her left parotid gland and subsequent histological examination confirmed a mantle cell lymphoma (common variant) relapse.

**Conclusion:**

Although rare, the involvement of parotid gland with mantle cell lymphoma must be considered in the differential diagnosis of parotid tumors.

## Introduction

Mantle cell lymphoma (MCL) is a rare lymphoma that accounts for approximately 5% to 7% of non-Hodgkin's lymphomas (NHL). It is usually characterized by an aggressive clinical course with a median overall survival of two to five years [1]. The involvement of extranodal sites is not uncommon as patients usually have an advanced-stage disease at the time of diagnosis [2]. According to Argatoff *et al*., the isolated extranodal location of the disease has been referred in 25% of cases, whereas salivary glands are rarely affected (only 3% of reported cases) [1]. Here we present a case of MCL of the parotid gland. We have also correlated its appearance at MRI with its histological findings.

## Case presentation

A 72-year-old Caucasian woman was referred to the Department of Haematology, General Hospital G. Papanikolaou, with a painful left parotid enlargement but without any other physical findings. Seven years prior to this presentation, she was diagnosed with MCL in her left submandibular gland, which was treated with surgical removal, radiotherapy, and a chemotherapy regimen of cyclophosphamide, hydroxydaunorubicin (Adriamycin), Oncovin (vincristine) and prednisone/prednisolone (CHOP). Her white blood cell (WBC) count of 5100/μL was represented by a normal differential of 69% neutrophils, 25% lymphocytes (with normal morphology), 5% monocytes and 1% eosinophils. Her hemoglobin, hematocrit and platelet levels were normal at 12.7 g/dL, 38.4% and 219/μL, respectively. Her bone marrow smears and biopsy were both normal without evidence of any infiltration by lymphoma cells.

**Figure 1:**
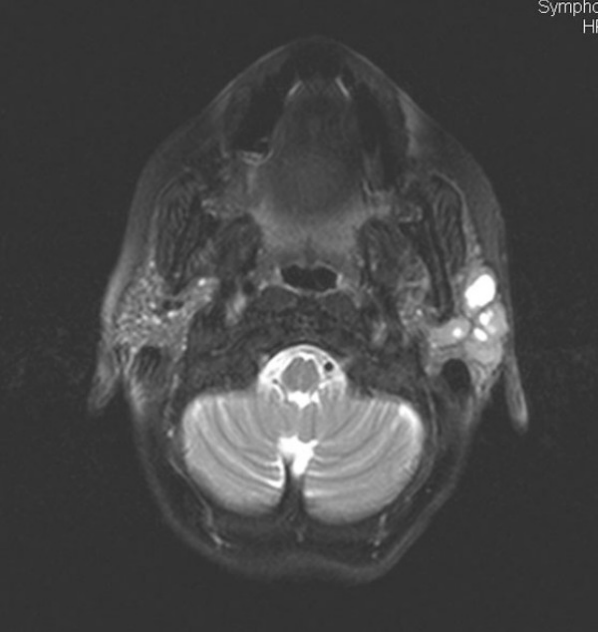
**Axial T2-weighted image reveals an enlargement of the left parotid gland and a total replacement of the normal parenchyma with mixed-type solid and cystic lesions**.

**Figure 2:**
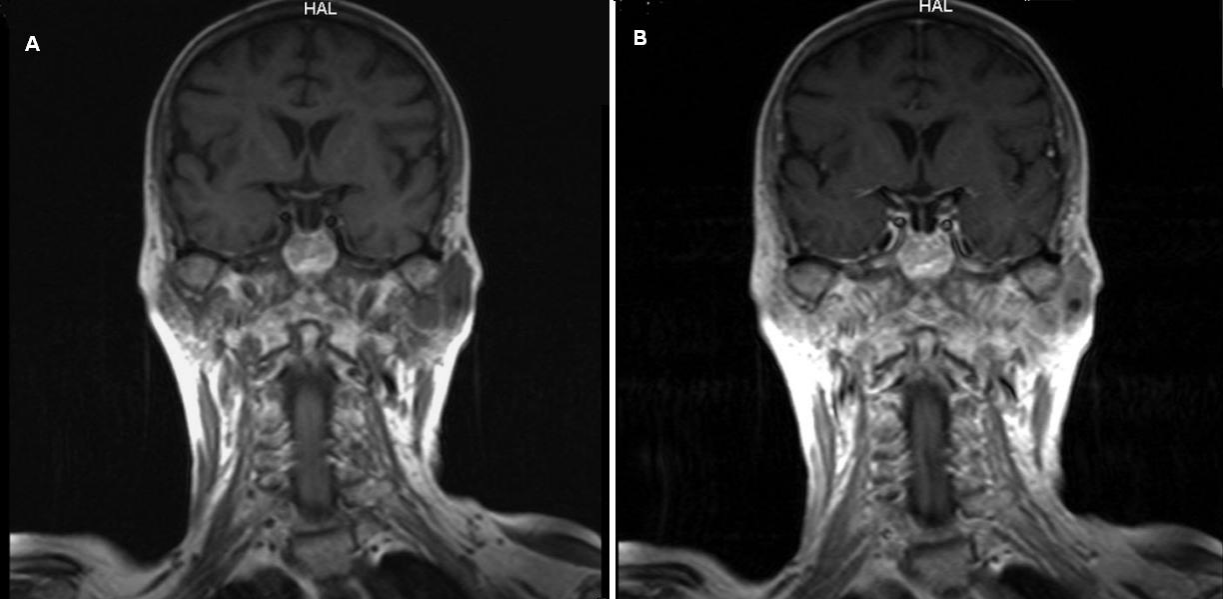
**(A) Coronal T1-weighted image shows the inhomogenic appearance of the left parotid gland**. (B) Coronal T1-weighted image after gadolinium contrast injection shows mild enhancement of the solid components, while the cystic areas remain hypodense.

An ultrasound examination of our patient revealed multiple cystic lesions in the parenchyma of her left parotid gland. Her MRI showed an enlargement of her left parotid gland and the total replacement of her normal parenchyma with mixed-type solid and cystic lesions (Figure 1). The solid components were mildly enhanced after an intravenous administration of contrast medium (Figures 2A and 2B). A whole body computed tomography (CT) scan showed the absence of pathologically enlarged lymph nodes. All the other organs of her chest and abdomen were also found to be normal.

**Figure 3:**
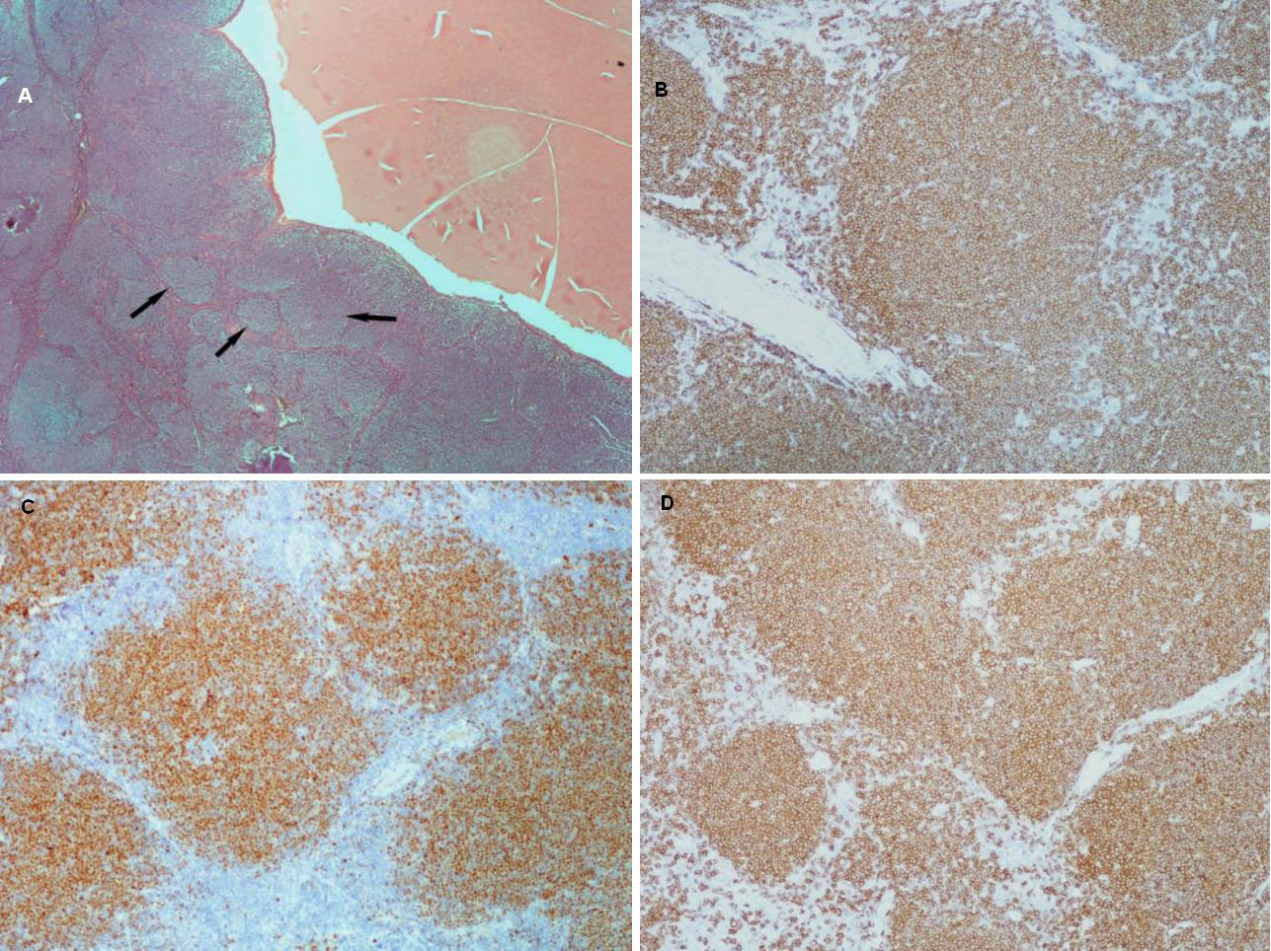
**(A) Microscopic appearance shows prominent vaguely neoplastic nodules (arrows) which are adjacent to the cystic area (red area) (hematoxylin and eosin staining, ×25 magnification)**. (B) Immunohistochemical stains for CD20, (C) CD5, and (D) cyclin D1 are positive (×100 magnification).

A biopsy of her left parotid gland and subsequent histological examination showed the presence of MCL (common variant). The MCL was composed of monomorphous small to medium-sized lymphoid cells which most closely resembled centrocytes with a vaguely nodular growth pattern. The prominent neoplastic nodules were adjacent to the cystic area (Figure 3A).

Immunostaining results for CD20, CD79a, CD43, CD5, sIgM(λ+) and cyclin D1 were positive (Figures 3B, C and 3D) and negative for CD23 and CD3.

Our patient was treated with partial parotidectomy and chemotherapy. Her post-operative course was uncomplicated. Eighteen months after surgery she was asymptomatic and disease-free.

## Discussion

Mantle cell lymphoma is a relatively rare and aggressive form of NHL. In the past, it has been referred to with various names including intermediately differentiated lymphocytic lymphoma, centrocytic lymphoma, and mantle zone lymphoma [1]. MCL has a characteristic morphological appearance consisting of small lymphoid cells with slightly irregular nuclear cells. Its histological growth patterns are of a nodular or diffuse type, or a combination of these two types [2,3]. In nodular MCL, some or many of the nodules may consist of follicles with reactive germinal centers surrounded by broad mantles of small lymphoid cells or the so-called mantle zone pattern [2]. Later in the course of the disease the mantle zonal or nodular pattern progresses to a diffuse pattern [4].

The immunohistological features of MCL reveal a characteristic phenotype. The cells express relatively intense surface immunoglobulin M (IgM) and/or immunoglobulin D (IgD) and are usually positive for CD5, FMC7 and CD43, but negative for CD10 and BCL6. CD23 is negative or weakly positive. Almost all cases of MCL are moderately to strongly positive for cyclin D1. Cyclin D1 expression can be detected in a subset of cases of chronic lymphocytic leukemia (CLL) and/or small cell lymphoma (SLL) and hairy cell leukemia. Usually, cyclin D1 is weakly positive in these neoplasms. Cyclin D1 can also be expressed strongly in approximately one-quarter of plasma cell myeloma cases.

Conventional cytogenetic analysis demonstrates a translocation between the immunoglobulin heavy chain and cyclin D1 (CCND1, BCL1 and PRAD1) genes, t(11;14)(q13;q32) in 70% to 75% of cases. However, almost all cases demonstrate the rearrangement of these genes using fluorescence *in situ *hybridization (FISH) probes [5]. This translocation can rarely occur in other types of B-cell NHL, in lymphocytic leukemia, and in multiple myeloma [2]. Therefore, cytogenetic findings need to be correlated with its pathological and immunological features to confirm a diagnosis of MCL.

Clinically, MCL occurs in middle-aged to older individuals with a median age of about 60 years, and predominantly in men [2]. Most patients present with a stage III or IV disease. According to Argatoff *et al*., in a clinicopathological study of 80 cases, extranodal involvement at presentation occurred in 76% of cases and the most common sites were the bone marrow (63%), peripheral blood (34%), gastrointestinal tract (10%) and Waldeyer's ring (10%). In 25% of cases the extranodal location was the disease's primary presentation, while the most common sites were the Waldeyer's ring (6%), intestine (5%), orbit (3%) and salivary gland (3%) [1].

MRI is the method of choice when treating patients with palpable parotid gland masses to assess the exact extent of the tumors, the invasion of neighboring structures, perineural spread, and lymph nodes staging [6]. Most parotid tumors, whether benign or malignant, rarely replace the parenchyma of the gland totally. Moreover, diffuse infiltration is often seen in lymphomas. The lymphomas have a homogenous signal pattern with low intensity on T1-weighted and high intensity on T2-weighted sequences. Although this MRI pattern is highly suggestive of the lymphoma, there is no absolute correlation between the imaging morphology and the histology of the lesion [7].

In this case, our patient's MRI showed an enlargement of her left parotid gland and a total replacement of her normal parenchyma with mixed-type solid and cystic lesions. The solid components were mildly enhanced using contrast medium. This appearance reflected the histological pattern of the lesion as MCL was composed of neoplastic nodules which were adjacent to the cystic area. To the best of our knowledge, there are no other published radiological findings on MCL of the parotid gland.

The differential diagnosis includes benign and malignant parotid tumors, especially Warthin tumors and adenoid cystic carcinomas, which may also have a solid cystic appearance. These tumors rarely occupy the total gland parenchyma. In particular, Warthin tumors are bilateral in up to 10% of cases reported. They present as well-circumscribed, partly cystic and partly solid lesions on MRI and are often located in the tail of the parotid gland. Adenoid cystic carcinoma usually presents as an infiltrating mass with a high propensity for perineural invasion. On MRI adenoid cystic carcinoma has an irregular contour, poorly defined margins, and a strong enhancement after the administration of contrast medium [6].

Special caution is required in the follow-up examination of patients with primary Sjögren's syndrome, as the risk of developing a lymphoma is increased. In this case the typical inhomogeneous nodular MRI picture seen in Sjögren's syndrome will change into a homogeneous pattern that can involve the parenchyma partially or even entirely [7].

## Conclusion

Isolated parotid gland involvement by MCL is very rare but should be considered nonetheless in the differential diagnosis when replacement of a patient's normal parotid parenchyma with mixed-type solid and cystic lesions is involved.

## Abbreviations

CHOP: cyclophosphamide, hydroxydaunorubicin (Adriamycin), Oncovin (vincristine) and prednisone/prednisolone; CLL: chronic lymphocytic leukemia; CT: computed tomography; Ig: immunoglobulin; MCL: mantle cell lymphoma; NHL: non-Hodgkin's lymphoma; SLL: small cell lymphoma; WBC: white blood cell.

## Consent

Written informed consent was obtained from the patient for publication of this case report and any accompanying images. A copy of the written consent is available for review by the Editor-in-Chief of this journal.

## Competing interests

The authors declare that they have no competing interests.

## Authors' contributions

MP performed the chart review and prepared the manuscript. AA evaluated and treated our patient, and also helped prepare the manuscript. FI was the pathologist who examined the specimens from our patient. TK and PP participated in manuscript preparation. All authors read and approved the final manuscript.
